# Melanocortin 1 receptor (MC1R) expression as a marker of progression in melanoma

**DOI:** 10.21203/rs.3.rs-3314825/v1

**Published:** 2023-09-19

**Authors:** David Su, Dijana Djureinovic, David Schoenfeld, Bernadette Marquez-Nostra, Kelly Olino, Lucia Jilaveanu, Harriet Kluger

**Affiliations:** 1 Division of Surgical Oncology, Yale University School of Medicine, New Haven, CT; 2 Division of Medical Oncology, Yale University School of Medicine, New Haven, CT; 3 Department of Radiology, Division of Advanced Medical Imaging Research, University of Alabama at Birmingham, Birmingham, AL

**Keywords:** MC1R, therapeutic targets, diagnostic markers, theranostics, progression markers

## Abstract

Melanocortin-1 receptor (MC1R) plays a critical role in human pigmentation and DNA repair mechanisms. MC1R-targeting agents are being investigated in clinical trials in melanoma patients, yet large studies investigating the rate and degree of MC1R expression in primary and metastatic human melanoma tissue are lacking. Using tissue microarrays containing three large cohorts of 225 cases of benign nevi, 189 with primary melanoma, and 271 with metastatic melanoma, we applied quantitative immunofluorescence and immunohistochemistry to comprehensively study MC1R protein expression. We show a stepwise elevation of MC1R expression in different stages of melanoma progression (nevi, primary, metastasis). Higher MC1R expression was seen in deeper (>1 mm) primary lesions, ulcerated lesions, and mucosal melanomas compared to cutaneous melanomas and was associated with shorter survival in primary and metastatic tumors. On multi-variable analysis, Breslow thickness, ulceration, male sex, and chronic sun exposure were independent predictors of worse overall survival in the primary melanoma cohort. In the metastatic melanoma cohort, MC1R expression and mucosal melanomas were independent predictors of inferior overall survival. Our data suggest that MC1R might be a valuable drug target in aggressive melanoma. Additional studies are warranted to determine its functional significance in melanoma progression and its utility as a predictive biomarker in patients receiving MC1R-directed therapies.

## Introduction

Cutaneous melanoma is the foremost cause of skin cancer-related fatalities, accounting for over 7,000 deaths annually in the United States.^1^ Moreover, the incidence of melanoma is increasing worldwide.^2^ For early-stage, localized melanomas, surgical resection is the mainstay of treatment. However, once the disease metastasizes or becomes unresectable, patients require systemic therapies such as chemotherapy, targeted therapy, or immunotherapy.^3^

Melanocortin 1 receptor (MC1R) is a transmembrane G-protein-coupled receptor protein found in melanocytes in the basal layer of the epithelium.^4^ MC1R interacts with its endogenous ligand alpha-melanocyte-stimulating hormone (aMSH) to activate the cAMP signaling pathway and *MITF* expression and to regulate melanin synthesis. Whole exome sequencing has shown MC1R to be highly polymorphic with over 200 nonsynonymous genetic variants in nearly all structural regions of the protein.^5^ While an increased prevalence of MC1R polymorphisms has been noted in cases of melanoma compared to controls, there is significant heterogeneity in the risk for melanoma across the many variants. Certain loss-of-function MC1R variants that correlate with phenotypic traits such as red hair and pale skin have demonstrated increased susceptibility to melanoma.^6^ In fact, melanomas from individuals carrying disruptive germline MC1R variants associated with red hair and freckling exhibit a higher somatic mutational burden than those without.^7^ Studies also show associations between MC1R variants and other genes implicated in melanoma, such as the synergistic effect of particular MC1R polymorphisms in patients with a germline CDKN2A mutation on melanoma risk.^8^ MC1R variants have been strongly associated with BRAF-mutant melanoma in non-chronic sun-induced damage melanoma.^9^

MC1R expression is known to be elevated in malignant melanoma as well as other types of skin cancers.^10^ While its native expression is restricted to melanocytes, MC1R can also be expressed on neurons, astrocytes, and microglia in the brain, and its activation has attenuating effects on neuroinflammation during traumatic brain injuries.^11^ High levels of MC1R transcripts have also been reported in a wide range of immune cells including helper T cells, natural killer cell subsets, CD14+ monocyte cell lines, B-cells, cytotoxic CD8+ T-cell subsets, and neutrophils.^12^ Moreover, MC1R activation by binding aMSH downregulates CD86 expression in CD14+ monocytes and reduces neutrophil chemotaxis.^13^ Furthermore, cytotoxic T-cell lymphocytes specific for MC1R-derived peptides were identified in tumor-infiltrating lymphocytes.^14^ Overall, MC1R expression is thought to be relatively limited to melanocytes, potentially making it a good target for therapies that enable cell destruction.

In addition to having an important role in regulating melanocyte pigment production, MC1R is a critical determinant of genomic maintenance and DNA repair. Binding of aMSH to MC1R may be functionally protective against UV-induced DNA damage as aMSH pretreatment of melanocytes can reduce generation of UV-induced oxidative byproducts, an effect that was absent in melanocytes expressing loss-of-function MC1R, suggesting the mechanism mediating this susceptibility may be independent of pigmentation.^15^ Additionally, aMSH has been shown to significantly inhibit growth of wild type MC1R transfected melanoma cell lines and reduce cell binding to extracellular matrix proteins compared to variant MC1R clones.^16^ Several studies have investigated a pigmentation-independent role of MC1R in UV-induced DNA damage repair. Swope et al noted that MC1R downstream signaling activates DNA damage sensors such as ataxia telangectasia mutation (ATM) and modulates the protein levels of xeroderma pigmentosum (XPC), which is critical in nucleotide excision repair.^17^ In human-derived melanoma cell lines, *MC1R* knockdown has been shown to significantly reduce survival and impair DNA repair mechanisms in response to UV light radiation compared to cells transfected with a shRNA control, increasing the risk of tumorigenesis.^18^ A study by Chen et al showed that rescuing MC1R loss-of-function in mice by drug-induced palmitoylation of MC1R can prevent melanomagenesis.^19,20^

Owing to the relative specificity of its expression, MC1R has garnered interest as a potential radiopharmaceutical target for theranostics, the pairing of diagnostics with targeted therapy.^21,22^ Conventionally, melanoma metastases are detected through noninvasive whole-body imaging techniques such as computed tomography (CT), magnetic resonance imaging (MRI), single-photon emission computed tomography (SPECT), and positron-emission tomography (PET).^23^ In current practice, [^18^F]fluorodeoxyglucose ([^18^F]FDG) is employed to assess both the metabolic and structural attributes of metastases. However, [^18^F]FDG is limited by its restricted utility in detecting micrometastases and metabolically inactive lesions. Furthermore, since the targeting ability of [^18^F]FDG is linked to glucose metabolism, [^18^F]FDG binding may be non-specific in the setting of catabolic diseases and inflammation and is less sensitive to indolent cancers. Consequently, MC1R is being explored as a melanoma-specific diagnostic probe.^24^

Recently, clinical trials are attempting to assess the effectiveness and safety of MC1R as a radiopharmaceutical target for metastatic melanoma.^25,26^ As with other targeted therapies, the efficacy of these agents relies on the expression of the target within the patient population affected by the disease. Previous studies have investigated MC1R protein expression in human-derived melanoma cell lines^10^ and formalin-fixed paraffin embedded tissue using mass spectrometry-based proteomics^27^ and immunohistochemistry^28^. However, these were significantly limited by relatively small patient cohort sizes. Therefore, we sought to determine expression patterns of MC1R in a large cohort of primary or metastatic melanomas and benign nevi using quantitative immunofluorescence to determine expression patterns and intensities.

## Results

**Figure 1.**
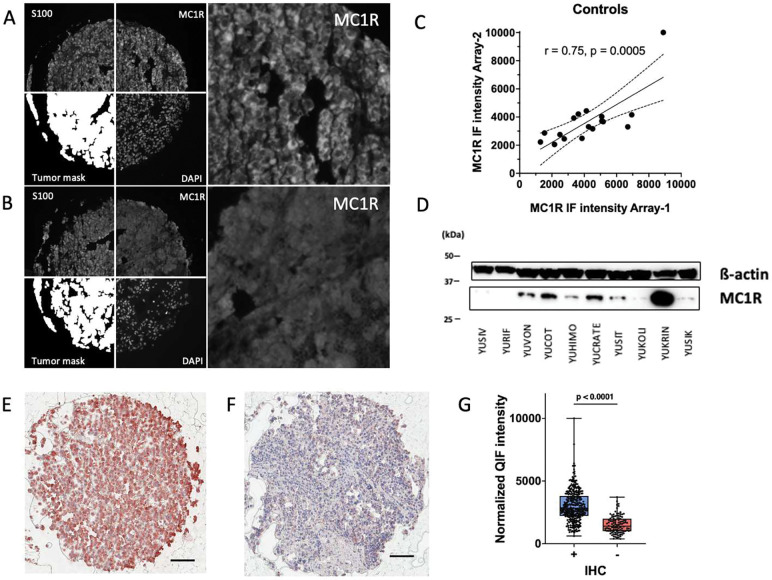
Expression patterns of MC1R using quantitative immunofluorescence (QIF) measurements of protein levels. MC1R expression is measured within the tumor mask (S100/PMEL) and nuclear DAPI stain. Examples of a histospot with strong MC1R staining (A) and weak MC1R staining (B). (C) Overlapping cases on the two arrays demonstrated a strong correlation (r = 0.75, p = 0.0005). (D) MC1R expression by Western blot was variable in intensity. Protein gel loading was assessed by ß-actin. YUSIV, YURIF, YUVON, YUSIT, YUKOLI, YUKRIN, and YUSIK were derived from patients with metastatic melanoma. Representative images of MC1R expression in patient tumor samples using immunohistochemistry (IHC) for MC1R+ (E) and MC1R- (F) tumors. Scale bar: 50 μm. (G) Box-and-whisker plot highlighting differences in continuous QIF intensities between MC1R positive versus negative cases by IHC. (p < 0.0001)

MC1R expression by immunofluorescence was assessed in two separate TMAs: one consisting of 189 primary tumors and 271 metastases, while the second contained 225 benign nevi. Figure 1A and 1B show examples of strong and weak immunoreactivity of MC1R in two representative histospots. MC1R antibody staining demonstrated strong correlation in individual tumors across established controls between the nevus and melanoma array (r = 0.75, *p = 0.0005*, Figure 1C) and good correlation between two replicate tissue microarrays (Supplementary Figure S1). Using Western blot analysis in a panel of melanoma cell lines, expression of MC1R was elevated in YUKRIN and YUCOT cells derived from metastatic tumors, and expression was variable with four cell lines demonstrating little to no expression (Figure 1D). MC1R expression was also assessed for positive vs. negative reactivity rather than degree of positivity in patient tumor samples with immunohistochemistry using AEC red substrate, with representative images depicted in Figure 1E and 1F. Notably, cases that were positive by IHC staining exhibited significantly higher QIF intensities than those that were negative by IHC (p < 0.0001), as highlighted in Figure 1G, supporting use of QIF for in depth interrogation of MC1R levels.

**Figure 2.**
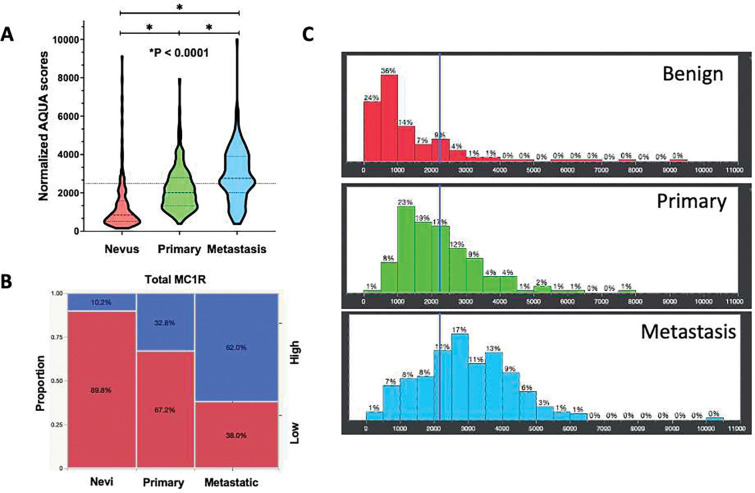
Expression patterns of MC1R in nevi, primary melanomas, and metastatic melanomas. (A) MC1R expression was higher in primary melanomas compared to benign nevi (*p < 0.0001*) and higher in metastases than in primary samples (*p < 0.0001*). The horizontal line denotes the cutpoint differentiating between “high” versus “low” MC1R expression using the median expression levels of malignant samples. (B) Mosaic plot illustrating MC1R high versus low expressing samples. (C) Histograms depicting tumoral immunofluorescence scores in benign nevi, primaries, and metastatic melanoma. The vertical line depicts the cut-point differentiating high versus low expressing samples.

Using quantitative immunofluorescence, we then tested the association between MC1R expression levels and disease progression. In primary melanomas, immunofluorescent intensity scores ranged from 395 to 7,936 with a mean and median score of 2,217 and 1,995 respectively, whereas in metastatic melanoma, scores ranged from 382 to 10,005 with a mean and median of 2,905 and 2,762 respectively. When comparing across benign nevi, primary, and metastatic melanomas, MC1R expression was significantly higher in primary melanomas than benign nevi (*p < 0.0001*) and higher in metastases than in primary sites (*p < 0.0001*), as shown in Figure 2A. To determine associations between “high” versus “low” MC1R expression, we dichotomized the continuous MC1R immunofluorescence intensity using the median score of all malignant melanomas as a cut-point, as there is no biological basis for dichotomization between high and low levels. Utilizing this threshold, 90% of nevi, 67% of primary melanomas and 38% of metastases had low MC1R expression levels (Figure 2B–C).

Clinical and pathological parameters were compared to continuous MC1R intensity scores in both the primary and metastatic melanoma cohorts using analysis of variance. In primary melanomas, we found a strong association between higher MC1R expression and a Breslow’s depth greater than 1 mm (*p < 0.0001*), presence of ulceration (*p = 0.0008*), presence of BRAF^V600E^ mutation (*p = 0.008*), and mucosal melanoma versus non-mucosal melanoma *(p = 0.0279*) (Table 1). Chronic sun exposure, age greater than 50, and male sex were not significantly different in MC1R expressing primary tumors. In the metastatic melanoma cohort, high MC1R intensity was not associated with age greater than 50, male sex, BRAF^V600E^ mutation or mucosal melanomas versus cutaneous melanomas.

**Figure 3.**
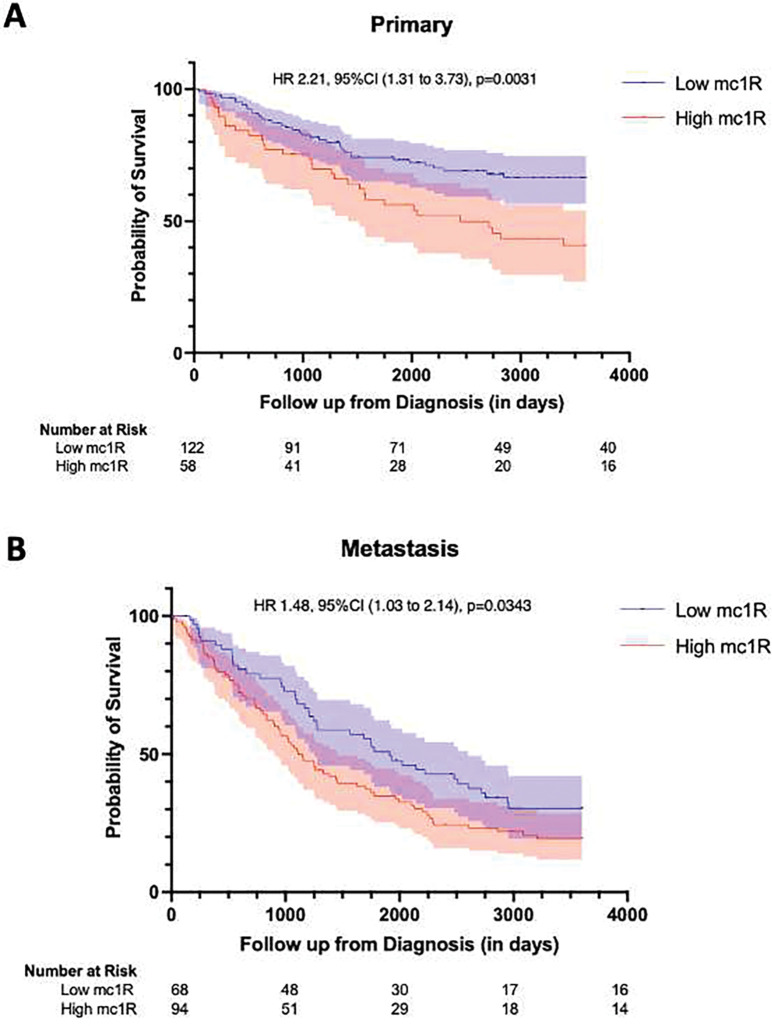
Kaplan-Meier curves in primary (A) and metastatic melanomas (B) cohorts by MC1R expression. High MC1R expression was associated with worse 10-year survival in the primary melanoma cohort (A) and metastatic melanoma cohort (B), *p = 0.0031* and *p = 0.0343* respectively. MC1R expression scores were dichotomized into “high” versus “low” using the median intensity score for all melanomas.

By Cox univariate survival analysis of continuous intensity scores, MC1R expression was strongly associated with decreased survival in primary tumors (*p = 0.009*) but was not a strong predictor of survival in metastatic specimens (*p = 0.1042*). (Supplementary Table S2) To visually assess the association between MC1R and survival, we dichotomized scores by the median value of all malignant melanoma and performed Kaplan–Meier analysis using log-rank statistics. Higher MC1R expression was associated with worse 10-year survival in primary melanomas (*p = 0.0031*) and metastatic melanoma (*p = 0.0343*). (Figure 3). On a multi-variable Cox proportional hazards analysis of primary specimens, thicker lesions (Breslow depth > 1 mm) were independently associated with worse survival (HR 2.6, *p=0.011*) as were presence of ulceration (HR 1.7, *p = 0.047*), male sex (HR 1.7, *p = 0.045*) and chronic sun exposure (HR 0.5, *p=0.017*) (Table 2). High MC1R expression was not found to be predictive in primary tumors. In the metastatic melanoma cohort, MC1R expression (HR 1.7, *p = 0.021*) and mucosal vs. primary melanoma (HR 2.4, p = *0.011*) were independent predictors of inferior overall survival. (Table 3)

## Discussion

We evaluated the expression of melanocortin 1 receptor (MC1R) in two distinct cohorts of patients, namely those with primary (stage I-III) melanoma and those with distant metastasis. We also interrogated a cohort of benign nevi. A quantitative approach utilizing immunofluorescence intensity measures was employed to measure MC1R expression levels in a fashion not discernable by standard IHC. We offer insight into the distribution of MC1R in melanoma progression and delineated a stepwise escalation in MC1R expression during progressive stages of melanoma, transitioning from benign nevi to primary melanoma to metastatic melanoma. Our study is unique as no prior studies have examined *in situ* MC1R levels in a large cohort of clinical specimens using a quantitative immunofluorescent method of expression analysis. This is especially significant in the context of the emerging treatments that target MC1R-expressing cells, where it is unknown whether there is an intensity threshold below which these approaches might not succeed.

The identification of prognostic and predictive biomarkers is paramount to patient stratification and effective melanoma management, supplementing traditional measures such as tumor thickness and ulceration in primary melanomas. The use of biochemical markers such as lactate dehydrogenase and S100 B protein for disease progression and prognosis has been well-documented. Furthermore, genetic markers such as BRAF and NRAS mutations can guide the use of targeted therapies and inform about disease behavior and response to targeted therapy.^29^ While it remains unclear whether MC1R overexpression is mechanistically important in melanoma, impaired MC1R function is linked to heightened skin cancer susceptibility and its variants have been explored as prognostic markers in metastatic melanoma and colorectal cancer.^30,31^ Our study underscores the potential of MC1R as a prognostic marker for death among patients with resected primary melanoma, a finding of particular relevance in the contemporary era with the wide adoption of adjuvant therapies. Markers of poor prognosis might enable us to preferentially treat those at higher risk of death.

From a clinical perspective, MC1R has remained an attractive therapeutic target because unlike other members of the G-protein family, MC1R is not highly expressed in most normal human tissues other than normal brain cells, melanocytes, and granulocytes, mitigating the concern for therapy-related toxicity.^32^ Furthermore, radiopharmaceuticals can be designed to bind specifically to MC1R protein as imaging agents to select patients likely to respond to MC1R-targeted treatments. Antibodies, antibody fragments, engineered proteins, and peptides are possible targeting vectors which can be developed to bind with high affinity to MC1R and labeled with radionuclides for noninvasive imaging using PET or SPECT. These imaging agents typically do not cross the blood-brain barrier hence there would be no uptake in normal brain cells unless the blood-brain barrier is compromised. Either way, noninvasive imaging of MC1R has the potential to guide clinical decisions for treatment.

While many studies have used the same antibody which binds a specific epitope of MC1R^33–35^, it is important to be mindful that MC1R in humans is highly polymorphic with over 200 documented germline and somatic variants. The most common mutations are observed in the intracellular and the transmembrane domains. However, potentially pathogenic variants can appear throughout the gene. Moreover, polymorphisms in the extracellular domain can lead to variations in structural conformations, and a truncating mutation in MC1R in the extracellular domains may result in an absence of detectable tumor protein expression.^36^ As such, antibodies would need to be strategically designed to bind to conserved regions of the extracellular domain which are less affected by genetic variants. Further studies should characterize the binding site of the MC1R protein, in the context of specific polymorphisms, and radiopharmaceuticals should be tailored to interact optimally with the various conformations of MC1R to ensure high binding affinity.

Several therapies targeting MC1R are being investigated in preclinical studies or clinical trials. One study showed that a single dose of [^225^Ac]Ac-DOTA-MC1RL for alpha-particle emitting therapy in mice bearing uveal melanoma xenografts had significantly prolonged survival and tumor growth delay while maintaining low *in vivo* toxicity.^21^ Another study showed that alpha-emitting particles remodel the irradiated tumor microenvironment ^37^ of treated mice and not only improved the mean survival time in treated mice but also increased the fractions of M1 macrophages, Th1 helper cells and activated natural killer cells in the TME, suggesting that combinations of these particles and immunotherapy may enhance treatment efficacy.^38^ Furthermore, MC1R-derived peptides induce a response from cytotoxic T lymphocytes (CTL) and tumor infiltrating lymphocytes (TIL), suggesting the potential for a MC1R-targeted melanoma therapeutic vaccine.^14^

Radiopharmaceuticals targeting MC1R are under investigation in patients with advanced melanoma (NCT05655312, NCT05496686).^21,25^ A phase I clinical trial is currently investigating the safety and efficacy of [^212^Pb]VMT01, an MC1R-targeting alpha-particle emitting agent, in patients with unresectable or metastatic melanoma.^25^ The study is designed as a dose-escalation and expansion trial involving up to 52 patients and seeks to determine the maximum tolerated dose of [^212^Pb]VMT01. Additionally, in a sub-study, a SPECT imaging surrogate [^203^Pb]VMT01, is being used to assess biodistribution and tumor uptake.^25^ [^203^Pb]VMT01 should mimic the pharmacokinetic properties of its therapeutic pair due to their similarity in chemical structure, thus dosimetry estimates for [^212^Pb]VMT01 would be more accurate than using other radionuclides for imaging. This study is currently accruing. Moreover, MC1R’s unique expression on the surface of melanoma cells may open up promising opportunities for development of targeted therapeutics including antibody-drug conjugates (ADCs) or cell therapies such as CAR-T therapies.

The association between high MC1R expression and worse survival in primary and metastatic lesions in this study may be multifactorial. Upregulation of DNA repair pathways in melanoma has been linked with metastasis and poor patient prognosis, which may be attributed to their ability to avoid catastrophic levels of DNA damage.^39^ It’s also not surprising to note that carrying certain MC1R variant alleles may confer a significantly increased risk of melanoma progression.^40^ Although most primary and metastatic melanoma specimens in our cohort had elevated MC1R expression, there remains a subset of samples with relatively low levels, and these patients might have poor response to MC1R-targeting therapies. Further work is required to pair this data with MC1R polymorphisms known to confer worse prognosis in melanoma and to assess the threshold below which MC1R-targeted therapies remains ineffective in melanoma tumors.

In summary, in light of recent approaches to target MC1R in melanoma, we demonstrate that MC1R expression is significantly higher in melanomas than nevi, making MC1R a target worthy further evaluation in radiopharmaceutical trials. Moreover, the association with poor survival in metastatic melanomas support further evaluation as a prognostic marker. Importantly, not all metastases expressed high levels of MC1R, suggesting that it might be important for patient selection. The association with aggressive disease substantiates the potential of MC1R-directed therapeutic strategies as a promising approach. Further work is required to determine whether there is an expression threshold below which responses to these therapies are not observed.

## Methods

### Patient cohort and tissue microarray construction

The melanoma and nevi tissue microarrays were constructed as previously described.^41,42^ Collection of patient specimens and clinical data was approved by the Yale University IRB. Paraffin-embedded, formalin-fixed tissue blocks were obtained from the Yale University Department of Pathology Archives and approved by Yale University Institutional Review Board. Representative regions of invasive tumor were examined by an experienced pathologist and 0.6 mm diameter cores were obtained from each specimen.

The melanoma array contains 230 primary melanomas and 293 metastatic melanomas resected between 1959 and 2000. Some spots had insufficient viable tumor tissue for analysis resulting in 189 tumors and 271 metastases respectively. 55% of patients were male, the mean follow-up was 6.7 years (range 2 months to 40 years), and the mean age at diagnosis was 52.4 years (range 18 to 91 years). The nevus array contains cores from 263 benign lesions as well as 40 metastatic or primary specimens from patients that were represented on the melanoma array.^43^ Both arrays contained identical cell lines, cored from pellets. Overlapping metastatic and primary specimens and cell lines were used for normalization of the scores obtained from the benign and malignant arrays.

### Immunohistochemistry and Immunofluorescent Detection of MC1R

Five μm TMA sections were mounted on glass slides and stained for MC1R. Staining was carried out as described previously.^44,45^ Briefly for immunofluorescence, slides were deparaffinized in xylene followed by two rinses in 100% ethanol. Antigen retrieval was done by boiling the slides in a pressure cooker containing Tris-ethylenediaminetetraacetic acid (pH 8.0, Santa Cruz Biotechnology, Dallas, TX, USA, sc-296654). Slides were incubated in methanol and 2.5% hydrogen peroxide for 30 min at room temperature to block the endogenous peroxidase activity. To block unspecific staining, slides were then incubated at room temperature for 30 min in 0.3% bovine serum albumin/1× TBS. Slides were incubated with the primary antibody (rabbit monoclonal anti–MC1R IgG; Abcam) diluted at 1:100 in TBS containing 0.3% bovine serum albumin at 4°C overnight. Slides were then washed thrice in 1× TBS/0.05% Tween 20. Goat anti-rabbit horseradish peroxidase (HRP)–decorated polymer backbone (Envision; Dako) was used as a secondary reagent. To create a tumor mask, slides were simultaneously incubated with a cocktail of mouse anti-S100 at 1:100 (ThermoFisher, clone 15E2E2) and mouse anti-HMB45 at 1:25 (BioGenex, MU001A-UC). For visualization of S100 and HMB45 staining, Cy3-tyramide (NED Life Science Products) was used. MC1R staining was visualized with Cy5-tyramide (NED Life Science Products). A nuclear mask was created by incubating the slides with 4,6-diamidine-2-phenylin- dole (DAPI, 1:200, Invitrogen, Carlsbad, CA).

For immunohistochemistry, slides were incubated with the primary antibody (rabbit monoclonal anti–MC1R IgG; Abcam) diluted at 1:25 in TBS containing 0.3% bovine serum albumin at 4°C overnight. The slides were washed thrice in 1x TBS/0.05% Tween 20 and incubated with an HRP-conjugated secondary antibody (Envision) for 2 hours at room temperature. After three TBS-T washes, 3-Amino-9-Ethylcarbazole (AEC) substrate solution (Abcam ab64252; red stain) was applied to the slide according to manufacturer’s instructions, and hematoxylin was used as a nuclei counterstain.

### Quantitative Determination of MC1R Expression

Image capturing and analysis were conducted using methods previously described.^45–47^ Tumor was distinguished from stromal elements via the use of S-100 and HMB45 signals to generate a tumor mask. A total tissue mask was defined using a DAPI+ nuclear compartment. Targets were visualized with Cy5. Tumor spots were excluded if they contained insufficient tissue (< 3% of the histospot area) or abundant necrotic tissue.

### Human cell lines

Yale University (YU)-designated cell strains were derived from primary and metastatic lesions as described.^48^ Ten low-passage (<20), patient-derived melanoma cell lines were obtained from the Cell Culture Facility of the Core of the Yale SPORE in Skin Cancer. These included YUVON, YURIF, YUSIV, YUKRIN, YUKOLI (lymph node metastasis), YUSIT, and YUSIK (lymph node metastasis). (Supplementary Table S1) All cell lines were maintained in 15-cm dishes and OptiMEM media (Invitrogen) supplemented with 10% heat-inactivated FBS (Invitrogen) and 1% antibiotic-antimycotic (penicillin, streptomycin, amphotericin B). Cells were incubated at 37°C in a humidified atmosphere of 95% air/5% CO_2_.

### Western blotting

Western blotting was performed by standard methods.^49^ Cells were lysed in NP40 solution supplemented with 1mM Na_3_VO_4_, 1mM PMSF containing the protease inhibitor cocktail. Protein concentrations of lysates were calculated by the BCA (Bicinchoninic Acid) assay. Protein (approximately 30 μg) was diluted in a sample buffer [2.5% SDS, 10% glycerol, 5% β-mercapto-ethanol, 50mM Tris (pH = 6.8) and 0.1% bromophenol blue] and subjected to sodium dodecyl sulfate-polyacrylamide gel electrophoresis (SDS-PAGE). A monoclonal rabbit anti-human anti-MC1R (Abcam) was diluted at 1:2000. β-Actin (1:10000, cat. # A5441, SIGMA) was utilized as a control for normalization of protein gel loading. Detection of proteins was done with peroxidase-conjugated anti-mouse (cat. # 7076S, Cell Signaling, 1:5000) or anti-rabbit IgG secondary antibodies (cat. # 7074S, Cell Signaling, 1:2000) and ECL (PerkinElmer).

### Statistical analysis

JMP Pro 16.2.0 software (SAS Institute, Cary, NC, USA), PRISM 9, and R were used for analysis. Data was analyzed using either continuous immunofluorescence scores or variables dichotomized at the median immunofluorescence score of malignant melanomas. The two-sample t test (analysis of variance) for continuous measurements and the Chi Square test for dichotomized variables were used to test relationships between MC1R expression levels and tumor type, site of metastasis, or intra-tumoral immune cell densities. Survival curves were generated using the Kaplan-Meier method. The association between continuous immunofluorescence scores and other clinical/pathologic parameters was assessed by ANOVA. The prognostic significance of parameters was assessed using the Cox proportional hazards model with survival as an endpoint.

## Supplementary Material

Supplement 1

## Figures and Tables

**Table 1.** Association between MC1R intensities in primary and metastatic melanomas and other clinical and pathological variables using continuous QIF scores. The association between MC1R expression and clinical variables was assessed by analysis of variance. Significance was determined using a p value of < 0.05.

MC1R expression and clinicopathologic features		
	Variable	P
Primary	Age at diagnosis (Age > 50)	0.6142
	Sex (Male)	0.2542
	Breslow’s depth (>1mm)	**< 0.0001**
	Ulceration present	**0.0008**
	Chronic sun exposure	0.3021
	Mucosal vs non-mucosal	**0.0279**
	Late stage (III/IV)	0.0741
	BRAF^V600E^ mutation present	**0.008**
Metastatic	Age at diagnosis (Age > 50)	0.528
	Sex (Male)	0.7147
	Mucosal vs non-mucosal	0.1529
	BRAF^V600E^ mutation present	0.1388

**Table 2:** Multivariate Cox proportional hazards model for primary samples using clinical and pathologic variables statistically significant in univariate analyses. MC1R expression level binarized by median cutoff score of malignant melanoma.

Multivariable Survival: Primary				
Variable	HR	Lower 95%	Upper 95%	P Value
Breslow > 1 mm	2.552	1.225	5.996	**0.011**
Chronic sun exposure	0.544	0.325	0.897	**0.017**
Male sex	1.669	1.012	2.794	**0.045**
Ulceration Present	1.679	1.008	2.776	**0.047**
High MC1R expression	1.565	0.928	2.619	0.092

**Table 3:** Multivariate Cox proportional hazards model for metastatic samples using clinical and pathologic variables statistically significant in univariate analyses. MC1R expression level binarized by median cutoff score of malignant melanoma.

Multivariable Survival: Metastatic				
Variable	HR	Lower 95%	Upper 95%	P Value
Mucosal melanoma	2.393	1.244	4.281	**0.011**
High MC1R expression	1.744	1.088	2.839	**0.021**
Age > 50	1.561	0.974	2.542	0.065

## Data Availability

Due to patient confidentiality and privacy concerns, all processed datasets generated for this study are available and deidentified from the authors upon request.
