# Supplementary material for: Melanocortin 1 receptor (MC1R) expression as a marker of progression in melanoma
Source: Res Sq. 2023 Sep 19:rs.3.rs-3314825. Preprint. [Version 1] doi: 10.21203/rs.3.rs-3314825/v1 (PMC10543287; doi:10.21203/rs.3.rs-3314825/v1)
Supplement: Supplement 1 [file NIHPPRS3314825v1-supplement-1.pdf]

The remaining authors declare that the research was conducted in the absence of any commercial or financial relationships that could be construed as a potential conflict of interest.

Supplementary Material

Sample/ passage	Gender/Age	Ethnicity	Stage	Tumor Location	Location of Primary lesion	Category	BRAF	NRAS
YUSIV 07-431	F/61	white	IV	metastasis to right chest	Right lower back, trunk	sun-exposed	PDE8A-RAF1 fusion	WT
YURIF 06-53	M/52	white	IV	recurrence right thigh	right thigh, leg	sun-exposed	V600K (AAG/GTG)	WT
YUVON 07-258	M/63	white	IV	recurrence right foot	acral right foot	acral	WT, multiple SNP/variations, synonym	4 changes polymorphism
YUCOT 09-804	F/33	white	IV	metastasis to ulna (long bone of forearm)	right neck	sun-exposed	V600E (GAG/GAG)	WT
YUHIMO 09-1215	M/58	white	IV	metastasis to right posterior thigh (large, amelanotic & raised)	acral, right heel	acral	PDE4DIP-BRAF fusion	WT
YUCRATE 13-2906	F/70	Hispanic	III	left lateral foot	acral, foot	acral	G469A-G	WT
YUSIT1	M/67	not available	IV	Unknown	metastasis	sun-exposed	V600K/WT (AAG/GTG)	WT
YUKOLI 08-518	M/53	white	III	metastasis to right axillary lymph node	right pectoral region, chest, trunk	sun-exposed	V600E/WT(GAG/GTG)	WT
YUKRIN 10-1692	F/37	white	IV	metastasis to brain (right frontal lobe)	left back, trunk	sun-exposed	WT (GTG)	WT
YUSIK 07-165	F/49	white	III+	metastasis to right inguinal lymph node	medial right thigh, leg	sun-exposed	V600E/WT (GAG/GTG)	WT

Supplementary Table S1. Human cell lines used for western blot analysis.

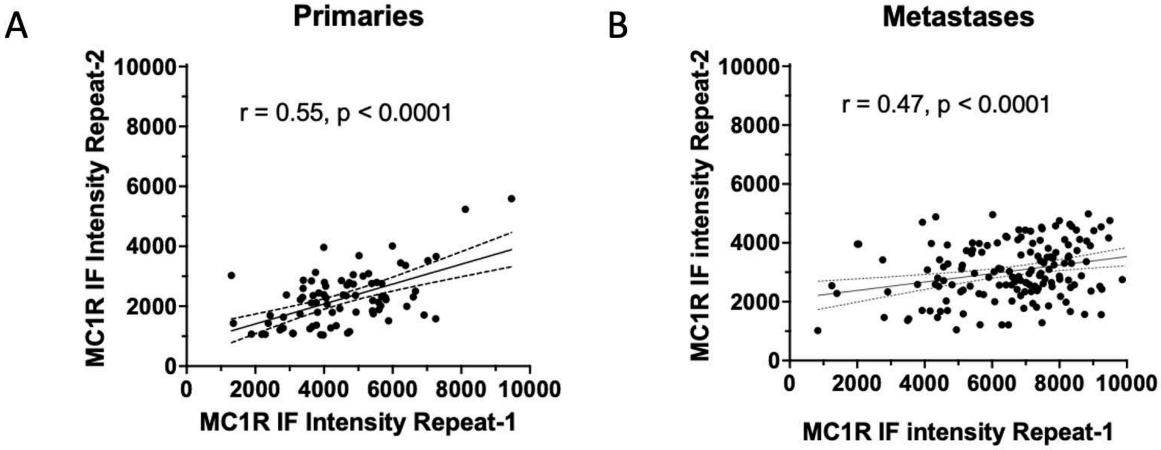

Supplementary Figure S1: Pearson's correlation comparing MC1R expression values between two replicate TMAs, showing strong relationship between overlapping primary (A) and metastatic (B) cases on the two arrays.

Univariate Survival Using MC1R intensity IF scores			
**Subset Type**	**Hazard ratio**	**95% C.I.**	**P-value**
Primaries	6.62	1.61-23.97	**0.009**
Metastases	1.86	0.78-3.91	0.1042

Supplementary Table S2. Univariate Cox Proportional Hazards model for primary and metastatic samples utilizing continuous MC1R intensity scores. Higher MC1R expression exhibits a worse prognosis in primary melanomas and trends toward worse survival in metastatic melanoma.
